# Books and Authors

**Published:** 1911-03

**Authors:** 


**Osteology and Syndesmology.** By **Howard A. Sutton,** A.M., M.D., Assistant in the Department of Anatomy of the University of Pennsylvania, and **Cecil K. Drinker,** B.S., 12 mo, pp. 225. Philadelphia: P. Blakiston's Sons & Co. 1910. (Cloth, $1.50.)

The difficulties of osteology are lightened in this clever little book. It is not overburdened with prosiness yet its scientific architecture is apparent from the first page. The labor of skipping from section to section as is necessary in the larger works, is removed here. The bones follow one another in simplest fashion and each joint is discussed after the bones which compose it. In this respect, if for no other, it is novel. It is wholly valuable and easily instructive.

---

**Non-Surgical Treatment of Duodenal Ulcer.** By **George Herschell,** M.D., London.

Dr. Herschell has reprinted in pamphlet form his paper on this subject, originally printed in the *Clinical Journal.* He does not take the stubborn stand that all cases of duodenal ulcer should be treated by medical means, irrespective of the patient's condition. He urgently recommends operation in the presence of marked interference of food passage, hemorrhage, or perforation, actual or impending. On the other hand, if the patient will permit medical treatment and conscientiously follow directions, he believes that many ulcers may be cured. His recommendations are interesting and based on common sense, apparently.

---

**Care of the Patient.** A Book for Nurses. By **Alfred T. Hawes**, A.M., M.D. 12 mo, pp. 173. With 6 illustrations. Philadelphia: P. Blakiston's Son & Co. 1911. (Cloth, $1.00, net.)

An unpretentious little book is this but full of valuable information for the nurse and directs to the safest and most satisfactory management of cases. It is not wholly intended for professional use; it will be of especial benefit to those who merely want a word of advice.

---

**A Manual of Pharmacy.** By **M. F. DeLorme,** M.D., Ph.G., Lecturer on Pharmacy and Pharmacology in Long Island College Hospital, New York. Second edition. 12 mo, pp. 207. With 19 illustrations. Philadelphia: P. Blakiston's Son's & Co. 1911. (Cloth, $1.25 net.)

The reception accorded the first edition of this interesting little work has necessitated a second issue. It has been improved in its reprinting. It is now as nearly perfect as possible a textbook on pharmacy for the practising physician and its reading and observance would be followed by improvement in the general use of drugs and in prescription writing.

---

**Hints for the General Practitioner in Rhinology and Laryngology.** By Dr. **Johann Fein,** Vienna. Translated by **J. Bowring Horgan,** M.B., B.Ch., London. 12 mo, pp. 239. Illustrated with 40 figures and 2 photographic plates. New York: Rebman Co. (Cloth, $1.50.)

A gem of a book which does not need the apology which the translator offers in his preface. It is a distinctly valuable contribution to the large number of smaller works which are intended for use more as aids than set textbooks. Yet it is one that is wholly understandable and convenient. It is full of helpful hints.

**The Practical Medicine Series.** Ten volumes. Under the general editorial charge of **Gustavus P. Head**, M.D. Vol. VIII. Pediatrics and Orthopedic Surgery. Edited by Isaac A. Abt, M.D., and John Ridlon, M.D. Vol. VIII. Therapeutics, Preventive Medicine, Climatology. Edited by George F. Butler, M.D., Henry B. Favill, M.D., and Norman Bridge, M.D.. Vol. IX. Skin and Venereal Diseases. Edited by W. C. Baum, M.D., and Harold N. Moyer, M.D. Vol. X. Nervous and Mental Diseases. Edited by Hugh T. Patrick, M.D., and John Ridlon, M.D. Series 1910. Chicago: The Year Book Publishers. (Price, $1.25 to $1.50; entire series, $10.00.)

In all these volumes the subjects have been carefully and exhaustively treated. All that is new in any of the departments of medicine has been gathered in orderly form. There is a marked improvement in the selections and arrangement of the material and as the publishers intend, the volumes form practically a complete résumé of the entire subjects under consideration.

---

**Publications of the Massachusetts General Hospital.** Vol. II, No.2. There are many interesting and instructive papers in this section of the reports of the Massachusetts General Hospital. The contents evince that sincerity and attention to detail which speak for the ultimate value of a scientific paper. There are twenty-two papers of exceptional merit in this volume, covering many interesting surgical and medical conditions.

---

**Principles of Public Health.** By **Thomas D. Tuttle,** M.D., Secretary of the State Board of Health of Montana. Yonkers: World Book Co. (Cloth, 50 cents.)

This is really a primer dealing with the commoner forms of health preservation. The author sets forth general rules, by observing which the human adult may do much to maintain his own health and so add to the effort toward the preservation of the public health.

---

**State Registration for Nurses.** By **Louie Croft Boyd,** R. N., Graduate of Colorado School for Nurses. 12 mo of 42 pages. Philadelphia and London: W. B. Saunders Company. 1911. (Price, 50c net.)

This pamphlet covers the laws governing the registration of nurses and the requirements necessary for a legal registration. It is in convenient form and is complete in every detail.

---

**Transactions of the Sixteenth Annual Meeting of the American Laryngological, Rhinological and Otological Society,** held in Washington, D. C., April 28, 29, and 30, 1910. Thomas J. Harris, M.D., Secretary.

A complete résumé of the sixteenth annual meeting of the American Laryngological, Rhinological and Otological Society held at Washington, D. C., for 1910. This book gives in their entirety the papers presented and the discussions.

**Progressive Medicine,** Vol. 12, December, 1910. A quarterly Digest of Advances, Discoveries and Improvements in the Medical and Surgical Sciences. Edited by **Hobart Amory Hare,** M.D., Professor of Therapeutics and Materia Medica in the Jefferson Medical College of Philadelphia, and Leighton F. Appleman, Instructor in Therapeutics at the same college. Philadelphia and New York: Lea & Febiger. (Per annum, paper bound, $6.00; cloth, $9.00.)

In this volume are the following papers: Diseases of the digestive tract and allied organs, the liver, pancreas, and peritoneum, by R. S. Lavenson; Diseases of the kidneys, by John Rose Bradford; Surgery of the extremities, shock, anesthesia, infections, fractures and dislocations and tumors, by Joseph C. Bloodgood; Genitourinary diseases, by William T. Belfield; Practical therapeutic referendum, by R. H. M. Landis.

---

**Primer of Hygiene.** By **John W. Ritchie,** Professor of Biology, College of William and Mary, Va., and Joseph S. Caldwell, Tenn. Yonkers: World Book Co. (Cloth, 40 cents.)

This is another of those small books which, with no pretense to stern scientific presence, gives a scientific subject that human treatment which brings it within the grasp of the commonplace lay mind. The principles of hygiene are laid down in such a manner as to be easy of grasp, and were its advice generally followed there would result much benefit to the average inhabitant.

---

**Lessons on the Eye.** By **Frank L. Henderson,** M.D., Ophthalmic Surgeon to St. Mary's Infirmary. Fourth edition. 12 mo, pp. 242. Illustrated. Philadelphia: P. Blakiston's Son & Co. 1910. (Cloth, $1.50.)

This is a small but serviceable book especially intended for the use of undergraduates and as such occupies a field essentially its own. It is comprehensively clear and the illustrations are decidedly helpful. Only a student lacking the essential elements of human intelligence would fail to get from its pages a working basis for the care of eye diseases and injuries.

---

**The Care and Training of Children.** By **LeGrand Kerr,** M.D., Brooklyn. 12 mo. New York: Funk & Wagnalls Company. (Cloth, 75 cents, net; by mail, 82c.)

The practising physician will find a great many useful and interesting facts in Dr. Kerr's book. He has condensed years' experience and observation as a physician, together with the more intimate observations of many parents who have made a study of the subject. He reflects in a series of monographs the matured experience of the many. There is scarcely a feature of child training, hygienic, physical, mental, or moral, that is not considered. It is a really worth-while book.

**State Board Examination Questions and Answers.** Third edition. Reprinted from the Medical Record. New York: William Wood & Co. 1910. (Cloth, $3.00.)

A third edition of the examination questions and the answers required by the various state boards of medical examiners has been issued. It is a well arranged book of 819 pages. Every physician or medical student who contemplates taking an examination should get a copy and make himself acquainted with its contents.

